# Indirect Estimation of Breathing Rate from Heart Rate Monitoring System during Running

**DOI:** 10.3390/s21165651

**Published:** 2021-08-22

**Authors:** Gaëlle Prigent, Kamiar Aminian, Tiago Rodrigues, Jean-Marc Vesin, Grégoire P. Millet, Mathieu Falbriard, Frédéric Meyer, Anisoara Paraschiv-Ionescu

**Affiliations:** 1 Laboratory of Movement Analysis and Measurement, École Polytechnique Fédérale de Lausanne, 1015 Lausanne, Switzerland; kamiar.aminian@epfl.ch (K.A.); tripleguitar26@gmail.com (T.R.); mathieu.falbriard@epfl.ch (M.F.); anisoara.ionescu@epfl.ch (A.P.-I.); 2 Applied Signal Processing Group, Institute of Electrical Engineering of the Swiss Federal Institute of Technology, École Polytechnique Fédérale de Lausanne, 1015 Lausanne, Switzerland; jean-marc.vesin@epfl.ch; 3 Institute of Sport Sciences, University of Lausanne, 1015 Lausanne, Switzerland; gregoire.millet@unil.ch (G.P.M.); frederic.meyer@unil.ch (F.M.)

**Keywords:** breathing rate (BR), heart rate (HR), RR intervals (RR_i_), respiratory sinus arrhythmia (RSA), frequency tracking

## Abstract

Recent advances in wearable technologies integrating multi-modal sensors have enabled the in-field monitoring of several physiological metrics. In sport applications, wearable devices have been widely used to improve performance while minimizing the risk of injuries and illness. The objective of this project is to estimate breathing rate (BR) from respiratory sinus arrhythmia (RSA) using heart rate (HR) recorded with a chest belt during physical activities, yielding additional physiological insight without the need of an additional sensor. Thirty-one healthy adults performed a run at increasing speed until exhaustion on an instrumented treadmill. RR intervals were measured using the Polar H10 HR monitoring system attached to a chest belt. A metabolic measurement system was used as a reference to evaluate the accuracy of the BR estimation. The evaluation of the algorithms consisted of exploring two pre-processing methods (band-pass filters and relative RR intervals transformation) with different instantaneous frequency tracking algorithms (short-term Fourier transform, single frequency tracking, harmonic frequency tracking and peak detection). The two most accurate BR estimations were achieved by combining band-pass filters with short-term Fourier transform, and relative RR intervals transformation with harmonic frequency tracking, showing 5.5% and 7.6% errors, respectively. These two methods were found to provide reasonably accurate BR estimation over a wide range of breathing frequency. Future challenges consist in applying/validating our approaches during in-field endurance running in the context of fatigue assessment.

## 1. Introduction

Recent advances in wearable technologies that integrate multi-modal sensors have enabled the in-field monitoring of several physiological metrics. In sport applications, wearable devices have been widely used to improve performance while minimizing the risk of injuries and illness [1,2]. Heart rate (HR) and heart rate variability (HRV) are undoubtedly the most accepted physiological metrics to follow health status and training load [3,4], while breathing rate (BR) is still underused [5]. BR is commonly assessed in the clinical field as an informative sign of physiological state [6] and one of the most sensitive vital indicator of clinical degradation [7,8,9,10]. Now, BR is also attracting interest in the field of sport science. Typically, BR might be used as a practical non-invasive method for evaluating ventilatory thresholds during graded exercise until exhaustion [5]. Moreover, it was shown that BR is closely associated with the subjects’ perceived exertion during an activity [5], as well as being sensitive to different fatigue states [11,12]. Furthermore, this physiological variable has a fast response at exercise onset/offset and changes rapidly in proportion to workload variations [5]. This makes BR a useful variable to monitor during training, as a marker for physical efforts and recovery.

Massaroni et al., 2019 displayed in their work, the different existing methods for measuring BR [13]. BR can be registered directly with portable devices that measure the flow-rate at the mouth. However, it requires the use of a facemask that is not well-adapted for in-field monitoring. With indirect methods, BR can be measured from the sound of breathing, the movements of the chest induced by respiration, or the modulation of biosignals induced by ventilation, such as electrocardiography (ECG) and photoplethysmography (PPG) signals. The majority of commercially-available wearable systems measure the BR from the thoracic and/or abdominal movement through strain sensors embedded into strap/chest belt or clothes [14,15,16]. However, these devices are relatively expensive and uncomfortable for the subjects. Thus, it is of great interest to provide robust and practical solutions for continuous and convenient monitoring of BR, by means of commonly used heart rate monitoring systems. 

Respiration affects ECG signals in several ways: baseline wander (BW), amplitude modulation (AM), and frequency modulation (FM) [17,18]. Respiration is known to modulate the heart rate such that it increases during inspiration and decreases during expiration, which is referred to as respiratory sinus arrhythmia (RSA). The RSA phenomenon has been widely explored and used in the clinical and psychological fields, mainly in resting conditions. It is well-accepted that RSA decreases with age and disease [19]. However, accurate data about RSA under exercise conditions are still scarce. The study of Blain et al., 2004 demonstrated that RSA and breathing dynamically developed at the same frequency during a graded and maximal exercise test [20]. Moreover, during exercise, spectral analysis of heart rate variability has highlighted that RSA represents the main mechanism regulating short-term heart rate (HR) fluctuations, particularly at very high intensities [21,22,23].

In the last twenty years, many researchers have investigated the possibility of deriving the BR by exploiting the influence of respiration on HR, ECG or PPG. This methodology necessitates two steps: (1) the extraction of respiratory signals from the original signal (ECG or PPG) through one of the modulations above-mentioned (BW, AM, FM independently or combined); and (2) BR estimation using various signal processing approaches. Pre-processing methods should be applied to remove long-range trends and non-respiratory frequency components.

Theoretically, BR can be estimated from the instantaneous frequency, computed by deriving the instantaneous phase of the signal, as given by the Hilbert transform [24]. However, the Hilbert transform yielded meaningful results only for narrow-band oscillations [25]. Other methods, such as Kalman filters [26] and energy tracking operators, can also be used to estimate the instantaneous frequency of a signal [24]. In real cases, particularly in biomedical applications, the above-mentioned methods do not seem to have found a widespread use because of their complexity and lack of robustness. Recently, Charlton et al., 2016 explored the performances of about 270 techniques to extract BR from ECG and PPG data in resting conditions. The main BR estimation techniques implemented were the Fast Fourier transform-based technique (FFT), auto-regressive spectral analysis, and time-domain approaches based on peak-detection [27]. It was found that all of the top-ranked algorithms for BR estimation at rest used time-domain breath-detection techniques [28]. Adaptive filters represent another type of approach to track the instantaneous frequency of a signal. In the context of atrial fibrillation detection, Buttu et al., 2013, presented two attractive adaptive filtering methods: single frequency tracking (SFT) and harmonic frequency tracking (HFT) for instantaneous frequency estimation [29]. 

To the best of our knowledge, only two studies extracted BR from heart rate or ECG data during physical exercises, and on a very limited sample. Mirmohamadsadeghi et al., 2016, performed a pilot study on two subjects during a VO2max test on an ergo-cyclometer using algorithms developed to analyze RSA at rest [30]. Lee et al., 2018, extracted respiratory rates from ECG data collected from 11 subjects with spontaneous breathing ranging from 36 to 60 breaths per minute (bpm) during treadmill exercises using a particle filter [31]. These two studies demonstrated promising results; however, they lack statistical robustness due to small sample sizes. 

Based on the existing background and developments, our study aimed to explore the performance of the promising BR estimation methods in the context of wide range of BR during physical effort. The main purpose was to compare and validate pre-processing and BR estimation algorithms using HR recorded with a chest belt during physical activities. The originality of this work is the analysis of RSA during running using a widely used HR monitoring system, yielding additional physiological insight without the need for an additional sensor. 

## 2. Materials and Methods

### 2.1. Data Set

A total of 31 healthy adult volunteers (22 males and 9 females, age 34 ± 10 years, size 174 ± 9 cm, weight 70 ± 12 kg) were asked to run at increasing speed until exhaustion on an instrumented treadmill (T-170-FMT, Arsalis, Belgium). The protocol was approved by the local ethical committee (CER-VD 2015-00006) and was conducted according to the declaration of Helsinki, and written informed consent was obtained from all the participants prior to the measurements. The test was designed with a speed that started at 8 km/h for 4-min and increased by 1 km/h every minute, followed by 1-min recovery. This incremental running test allowed us to measure a large BR range. RR intervals were measured using the Polar H10 heart rate monitoring system (ECG single-lead, sample rate 130 Hz, Polar^®^, Finland) attached on a chest belt (Polar ProStrap). The ground truth device that we used to evaluate the accuracy of the BR estimation algorithms was the Cosmed Quark CPET system (COSMED Srl, Rome, Italy). This metabolic measurement system allows a direct BR estimation using a facemask that covers the nose and mouth of the subject. Prior to each test, the Cosmed system was calibrated according to the manufacturer’s instructions. The systems were synchronized manually (i.e., starting the devices at the same time). 

### 2.2. Framework of the Breathing Rate Estimation from RR Intervals and Validation

**Figure 1:**
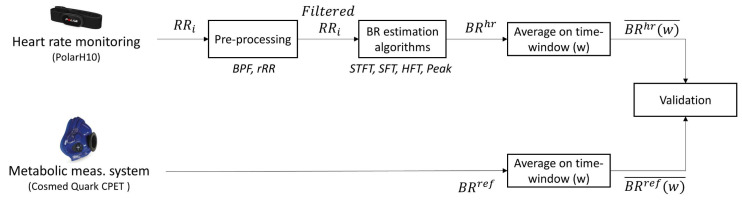
Flowchart of the method used for breathing rate (BR) estimation from RR intervals and validation procedure against the reference system. ${\mathrm{RR}}_{i}$: RR intervals; BPF: band-pass filter; rRR: relative RR intervals transformation; STFT: short-term Fourier transform; SFT: singular frequency tracking; HFT: harmonic frequency tracking; Peak: peak detection. $\bar{\mathrm{BR}}$ designates the averaged value of BR on the different window sizes (w) explored.

The framework developed in this study can be divided into four parts, as illustrated in Figure 1. The ‘Pre-processing’ step consisted of filtering the respiratory component from the RR interval (${\mathrm{RR}}_{i}$) time-series by removing non-respiratory frequency components (see Section 2.3). Then, BR was estimated from the obtained filtered ${\mathrm{RR}}_{i}$ signal using four BR estimation algorithms. Both the frequency and time-domain methods were evaluated; three algorithms were based on frequency-domain estimation and one was based on time-domain estimation. All combinations of pre-processing and BR estimation algorithms were evaluated. The third step consisted of averaging both the obtained BR estimation and the reference BR measurement on different window sizes from 10 s to 60 s. Finally, the two systems were compared to evaluate the performances of all combinations on the different time windows. These four steps are detailed in the following sections.

### 2.3. Pre-Processing Methods

In our study, we are working directly with the time interval between consecutive R-peaks, namely RR intervals (${\mathrm{RR}}_{i}$), where its frequency is modulated by respiratory signal. The initial pre-processing step, common to all techniques, was the resampling of RR intervals to 6 Hz using cubic spline interpolation [32] in order to ensure regular sampling [28]. Two pre-processing methods, inspired by previous work, were implemented to remove non-respiratory frequency components and highlight the respiratory oscillations: band-pass filtering (BPF) and relative RR intervals transformation (rRR). The BPF is commonly used and was applied to all techniques implemented by Charlton et al., 2016, in their review [27]. Then, as we expected the frequency modulation of the ${\mathrm{RR}}_{i}$ to reduce during running, we tried a new processing method for HRV assessment, which was developed by Vollmer [33]. This rRR transformation has not yet been explored in the current context, but we considered it as promising to enhance the respiratory oscillations. These methods are detailed in the following paragraph.

#### 2.3.1. Band-Pass Filtering (BPF)

BPF was used as a pre-processing method to eliminate non-respiratory frequencies by filtering the ${\mathrm{RR}}_{i}$ time-series with a band-pass filter from 0.2 to 1.2 Hz [28]. There is no consensus on the optimal range of plausible respiratory frequencies and it is necessary to adjust the filter parameters according to the population or application. At rest, a band-pass filter with −3 dB cutoff frequencies of 4 to 60 bpm is generally applied [27]. During the running test, the breathing frequency ranges over a large spectrum, from 10 to 70 bpm. Thus, we selected a Chebyshev type I band-pass filter with less than 0.5 dB of ripple in the passband defined from 0.2 to 1.2 Hz, and at least 60 dB of ripple in the stopband. This filter was chosen to ensure a fast roll off.

#### 2.3.2. Relative RR Intervals (rRR)

The second pre-processing method used in this study was the rRR transformation. This method considered the difference of consecutive RR intervals weighted by their mean (Equation (1)), which was relevant in our application, where the heart rate increased during the running incremental test. This new approach to investigate HRV based on relative RR intervals was suggested in [33].

(1) $$rr_{i}\;:=\frac{2\;\left(RR_{i}-RR_{i-1}\;\right)}{RR_{i}+RR_{i-1}}\;,\;i=2,\ldots ,\;n$$

where *n* is the total number of ${\mathrm{RR}}_{i}$. The sequence rr describes the relative variation of consecutive ${\mathrm{RR}}_{i}$, which is usually between ±20%. RR intervals of 1 s (heart rate of 60 beats per minute) have a variation range of about 0.1 s, i.e.,10%. The same variation of ±0.1 s would not be possible for a stress situation with RR intervals of 0.4 s (heart rate of 150 beats per minute). This is unphysiological and without relation to heart capacity/capability. Thus, the rRR transformation took into account the relative variation of consecutive ${\mathrm{RR}}_{i}$, contrary to the common HRV measures. This transformation directly removed the long-term trend in the ${\mathrm{RR}}_{i}$ time-series due to an increase in HR during the running incremental test. Next, a BPF was applied to remove non-respiratory frequency components. 

### 2.4. Breathing Rate Estimation

#### 2.4.1. Short-Term Fourier Transform (STFT)

The STFT is an adapted version of the Fourier transform to determine the frequency spectrum of local sections of a signal, which is appropriate for analyzing non-stationary signal. A long Hamming window of 91 s was selected to provide a suitable frequency resolution. Then, the frequency corresponding to the local maximum in the magnitude of the STFT was used as a BR estimation.

#### 2.4.2. Single Frequency Tracking (SFT)

**Figure 2:**
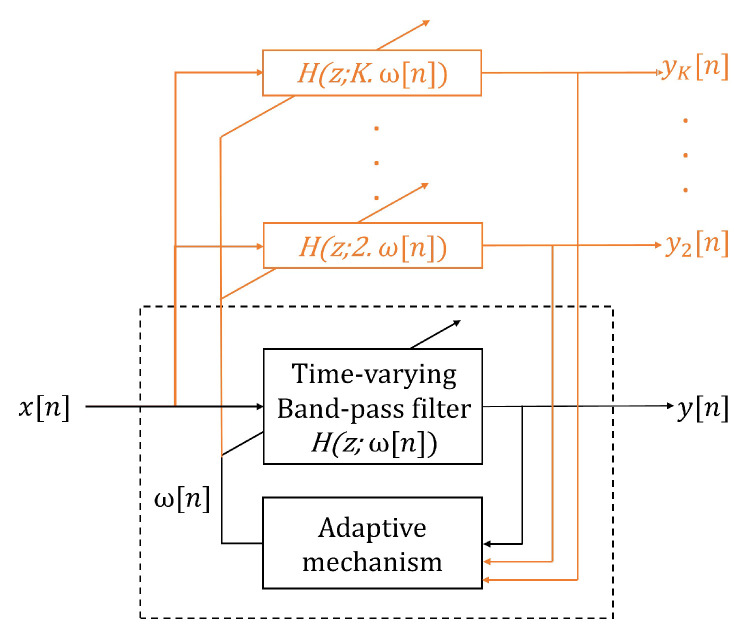
Adaptive band-pass filter: the dashed black square corresponds to the single frequency tracking (SFT) algorithm structure. SFT is composed of a time-varying bandpass filter, with a transfer function given by H(z, ω[n]), and an adaptive mechanism. x[n] and y[n] are the input and filtered output signal, respectively, and ω[n] is the estimated instantaneous frequency. The orange parts show the extension of the SFT structure to create the harmonic frequency tracking (HFT) algorithm. In this situation, $y\left[n\right]$ and $y_{k}\left[n\right]\;$ are the filtered output signals [29].

Another method that was tested to estimate the BR frequencies from the filtered ${\mathrm{RR}}_{i}$ is the SFT algorithm, which is based on an adaptive band-pass filter [29]. The SFT is composed of two parts: a time-varying bandpass filter and an adaptive mechanism (Figure 2). The output signal, y(n), is obtained by filtering the input signal, x(n), with the time-varying bandpass filter. In the current work, the input and output signals refer to the filtered ${\mathrm{RR}}_{i}$ and the ${\mathrm{BR}}^{\mathrm{hr}}$, respectively (Figure 1). The transfer function of the bandpass filter is given by:

(2) $$H\left(z;\omega \left[n\right]\right)=\frac{1-\beta }{1-\beta e^{j\omega \left[n\right]}z^{-1}}$$

ω[n] determines the central frequency of the filter and the parameter β (0 ≪ β < 1) controls the bandwidth. In this study, the bandwidth was set to β = 0.90. Thus, the adaptive mechanism was based on minimizing the mean square error of a discrete oscillator equation, which provided the estimation of the instantaneous frequency ω[n]. 

#### 2.4.3. Harmonic Frequency Tracking (HFT)

The HFT algorithm was also evaluated to extract the BR frequencies. This algorithm is an extension of the SFT that includes the tracking of the harmonic components. This extension consists of using one time-varying bandpass filter for the fundamental component and each harmonic component, as shown in orange in Figure 2. An adaptive mechanism with a weighting procedure was designed in order to compute a global estimate of the instantaneous fundamental frequency [29]. In this study, we implemented the HFT filter such as only the fundamental and the first harmonic component are tracked, because higher harmonics have a very limited amplitude. A detailed explanation of the SFT and HFT adaptive mechanisms are given in the work from A. Buttu et al., 2013 [29]. 

#### 2.4.4. Peak Detection (Peak)

The last BR estimation algorithm evaluated in this study was a time-domain based method, which combined peak and trough detection applied to the filtered RRi [34]. Peaks that were lower than the mean, and troughs that were larger than the mean, were removed. Then, a threshold of 0.5 s between each peak (and trough) was applied. Finally, only peaks that were immediately followed by a trough were considered as valid.

### 2.5. Validation Procedure

In this study, a breath-by-breath comparison could not be performed as the above-mentioned methods did not allow such estimation. In addition, BR obtained from gas exchange systems are commonly filtered to remove erratic breaths resulting from coughs, swallows, etc. [35]. Thus, the estimated and the reference BR data were averaged across 10 s, 20 s, 30 s, 40 s, 50 s and 60 s time-windows [28,31]. The agreement between the two datasets was assessed in terms of Bland–Altman statistics and the relative percentage error. 

The error *ϵ*(w) and the absolute percent error *ϵ%*(w) were defined as the difference between the wearable and the reference system for each window:

(3) $$\epsilon (w)=\bar{BR^{hr}}\left(w\right)-\bar{BR^{ref}}\left(w\right),$$

(4) $$\epsilon (w)=\frac{\left|\bar{BR^{hr}}\left(w\right)-\bar{BR^{ref}}\left(w\right)\right|}{\bar{BR^{ref}}\left(w\right)}*100,\;w:\;\mathrm{time}\;\mathrm{window}$$

As we did not focus on intra-subject variability, the overall bias and precision were computed by pooling error values of data from all athletes, with about 30 values per athlete depending on the window size [36]. Then, gaussian distribution of the errors ϵ(w) and *ϵ%*(w) was tested using the Shapiro–Wilk test and the normality was visually double checked using the Q–Q plot. In case of non-normally distributed errors, the median and interquartile range (IQR) were used as measures of bias and precision, respectively. Furthermore, the Bland–Altman statistics were plotted, showing the bias and 95% confidence interval (CI). The $2.5{\mathrm{th}}^{}$–$97.5{\mathrm{th}}^{}\;$ percentiles were used as an estimation of 95% CI for non-normal distributions [37]. 

Secondly, as a large range of BR was continuously assessed throughout the incremental running test, the relationship between $\bar{{\mathrm{BR}}^{\mathrm{hr}}}$ and $\bar{{\mathrm{BR}}^{\mathrm{ref}}}$ could be evaluated using their correlation coefficient. A linear regression was applied on the pooled BR values of all subjects across all exercise intensities. The non-parametric Spearman correlation was preferred to the Pearson correlation in cases of non-normally distributed data [38].

## 3. Results

Out of the 31 participants, 29 were kept for the evaluation of the proposed system. Three participants were removed because of missing data. An average VO2max and velocity at maximal oxygen uptakes (VMA) of 56.2 ± 9.1 mL/kg/min and 16.9 ± 2.8 km/h, respectively, were obtained from this database, and were directly computed using the Cosmed system. In addition, the minimum and maximum BR obtained from the reference system were 8.5 ± 2.4 bpm and 71.5 ± 15.6 bpm, respectively. 

### 3.1. Estimation of the BR from RR Intervals

#### 3.1.1. Pre-Processing

**Figure 3:**
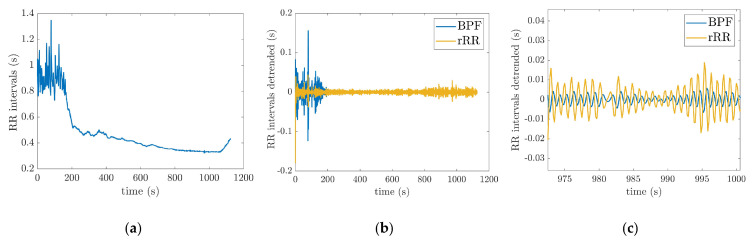
Pre-processing methods: (**a**) raw RR_i_ time-series of one subject during the incremental running test; (**b**) filtered RR_i_ obtained from raw RR_i_ using the two pre-processing techniques (BPF: band-pass filter; rRR: relative RR intervals transformation); (**c**) zoom in on (**b**) to illustrate the respiratory oscillations. The incremental running test started with a speed at 8 km/h for 4-min. Then, the treadmill speed was increased by 1 km/h every minute, followed by 1-min recovery.

Figure 3a shows raw ${\mathrm{RR}}_{i}$ time-series of one subject measured by the PolarH10 sensor during the test. In order to remove the long-range trend and non-respiratory frequency components, the ${\mathrm{RR}}_{i}\;$ time-series was filtered using the pre-processing methods described in Section 2.3 (Figure 3b). The zoom-in on Figure 3b, shown in Figure 3c, illustrates the oscillation due to respiratory signal after the processing of ${\mathrm{RR}}_{i}$. The distance between two positive peaks represents a breathing cycle (inhalation and exhalation). 

#### 3.1.2. BR Estimation Algorithms

**Figure 4:**
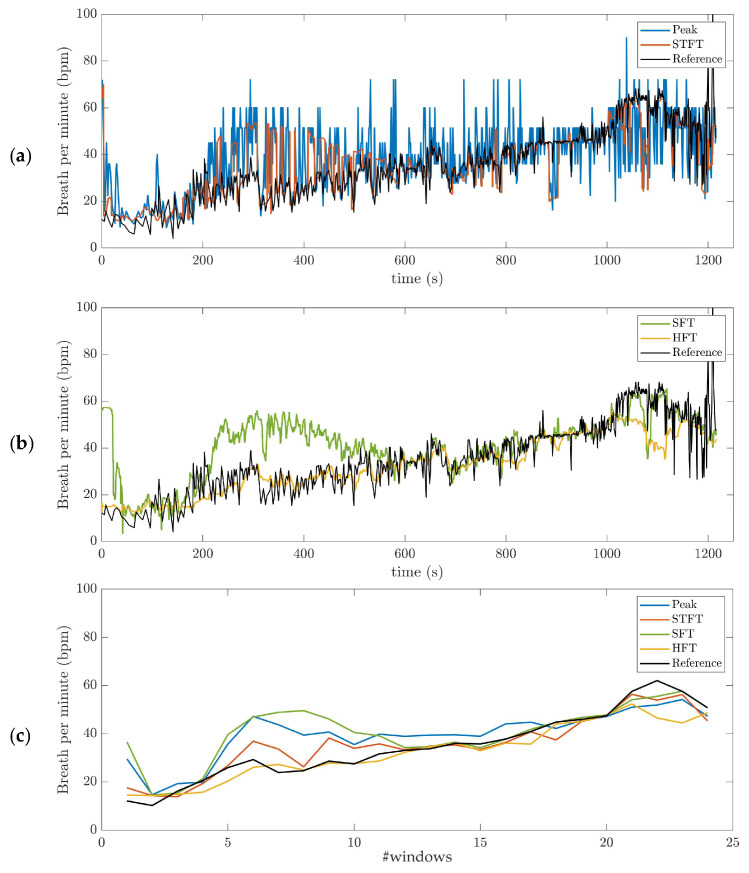
Breathing rate (BR) estimation, illustrated on data obtained for one subject during the incremental running test. The band-pass filter (BPF) (0.2–1.2 Hz) pre-processing method was used: (**a**) short-term Fourier transform (STFT) and peak detection (Peak); (**b**) Single and Harmonic frequency trackers (SFT and HFT); (**c**) average values obtained across 50 s time-windows for the four BR estimation algorithms (Peak, STFT, SFT and HFT), as well as the reference system. The dark line is the reference BR from Cosmed. The incremental running test started with a speed at 8 km/h for 4-min. Then, the treadmill speed was increased by 1 km/h every minute, followed by 1-min recovery.

A visual analysis of the instantaneous BR estimation provides interesting insights. Figure 4a,b shows the BR estimate in one subject using the four different algorithms. In this example, the ${\mathrm{RR}}_{i}\;$ time-series was filtered with the BPF (0.2–1.2 Hz) pre-processing method. Outliers are observed in BR estimation based on peak detection and STFT (Figure 4a). The BR estimates obtained using the adaptive filter SFT and HFT appear much smoother as compared to the other estimators (Figure 4b). In addition, HFT seems to have better performance than the SFT. It is worth mentioning that the ${\mathrm{BR}}^{\mathrm{ref}}$ from Cosmed system (dark line in Figure 4) is not filtered for comparison purposes. Finally, Figure 4c shows the average values obtained across 50 s time-windows for the four BR estimation algorithms (Peak, STFT, SFT and HFT), as well as the reference system. 

### 3.2. Validation

The performances of all possible combinations of pre-processing and BR estimation algorithms were evaluated, resulting in eight algorithms (Table 1). The Shapiro–Wilk test and Q–Q plot demonstrated that the errors *ϵ*(w) were not normally distributed (see Appendix A). Consequently, non-parametric statistical tests were used for evaluation. Table 1 lists the bias (median of the error *ϵ*(w)), interquartile range (IQR), CI ($2.5{\mathrm{th}}^{}$–$97.5{\mathrm{th}}^{}\;$ percentiles), median absolute percent error (MdAPE, median of *ϵ%*(w)) and Spearman’s correlation (ρ) obtained for each combination when BR is averaged across 50 s window durations. Results computed across 10, 20, 30, 40, 50 and 60 s window durations are provided in Appendix B. 

#### 3.2.1. BR Estimation Algorithms and Bland–Altman Plot

The results provided in Table 1 demonstrate that the pre-processing methods used to detrend and filter the raw RR intervals plays an important role in the accuracy of BR estimation. The classic BPF shows the best results for three out of four BR estimation methods (BPF + STFT: −0.08 (3.28) bpm; BPF + SFT: 1.31 (5.89) bpm; and BPF + Peak: 2.85 (8.27) bpm) compared to rRR pre-processing. Results obtained with rRR reveal a high positive bias and IQR for all combinations (rRR + STFT: 2.35 (13.22) bpm; rRR + SFT: 7.30 (17.20) bpm; and rRR + Peak: 9.18 (14.93) bpm), except when HFT is used (rRR + HFT: 1.06 (4.29) bpm). The histograms of the error ϵ(w), provided in Appendix A, confirm these results; the non-gaussian distributions with a long positive tail for rRR + STFT, rRR + SFT and rRR + Peak, indicate an important overestimation of the BR when rRR is used as a pre-processing method. However, rRR combined with HFT demonstrates interesting results that have the highest correlation with the reference system (ρ = 0.88, *p* < 0.05). As the BR increases throughout the running test, we also computed the absolute relative error ϵ%(w), which was divided by the magnitude of the reference value. Three BR estimation algorithms show a relative error below 8% (BPF + STFT: 5.48%, BPF + SFT: 8.10% and rRR + HFT: 7.66%).

**Figure 5:**
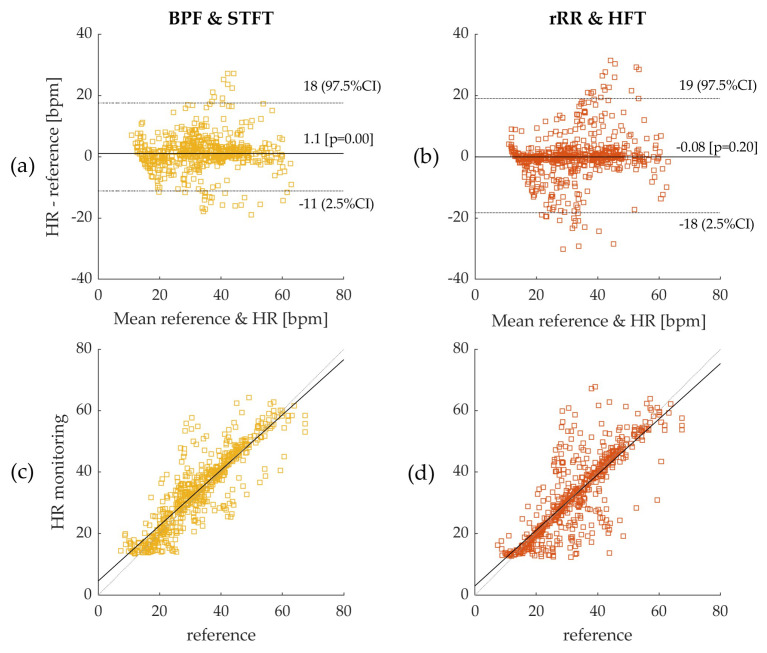
(**a**) Bland–Altman plot: Band-pass filter (BPF) and short-term Fourier transform (STFT); (**b**) Bland–Altman plot: relative RR intervals transformation (rRR) and Harmonic frequency tracking (HFT); as the distributions are non-gaussian, bias was computed using median and 95% confidence interval (CI) using $\;2.5{\mathrm{th}}^{}$–$97.5{\mathrm{th}}^{}\;$ percentiles. Errors were computed on 50s time-windows and pooled from all participants leading to a sample size of 609 values. Dotted lines on figures (**a**,**b**) represent 95% CI and solid line represents the bias (bpm); (**c**) Spearman’s correlation for BPF + STFT; (**d**) Spearman’s correlation for rRR + HFT. The reference system indicated in the figures corresponds to the Cosmed Quark CPET measurement device used as ground truth.

Furthermore, the Bland–Altman plots with 95% CI obtained for all methods are provided in Appendix C, with the results summarized in Table 1 (time-window of 50 s) and Appendix B (time-window of 10, 20, 30, 40, 50 and 60 s). Figure 5 shows the Bland–Altman and Spearman’s correlations plots for BPF + STFT and rRR + HFT selected as the best methods. The Bland–Altman plots indicated a bias of −0.08 bpm (95% CI: +19.1, −18.2 bpm) for BPF + STFT and 1.1 bpm (95% CI: +16.46, −12.24 bpm) for rRR + HFT (Figure 5a,b). The low systematic bias indicates good agreement with the reference system for both approaches. Interestingly, the ${\mathrm{BR}}^{\mathrm{hr}}$ errors and precision do not depend on the frequency range (Figure 5). Spearman correlation coefficients showed that the measurements by the two systems were consistently correlated overall with ρ-values of 0.82 (*p* < 0.05) for BPF + STFT and 0.88 (*p* < 0.05) for rRR + HFT (Figure 5c,d).

#### 3.2.2. The Effect of Time-Window Duration

**Figure 6:**
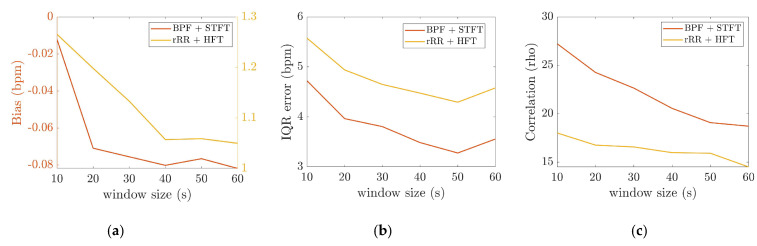
Effect of the time-window averaging computed for BPF + STFT and rRR + HFT, the *x*-axis corresponds to the different time-windows durations: (**a**) bias (bpm), as the two methods have different range of bias, left and right y axis are on two different scales for STFT and HFT, respectively; (**b**) interquartile range (IQR); (**c**) Spearman’s correlation.

In Figure 6, it can be observed that the IQR decreased as the time-window duration increased up to 50 s, from 5.5 to 4.3 bpm, and from 4.7 to 3.3 bpm for HFT and STFT, respectively. Accordingly, the correlation coefficients increased from 0.86 to 0.88, and from 0.74 to 0.82, for HFT and STFT, respectively, until a time-window of 40–50 s was reached. The bias also changed as a function of the window duration. However, differences of +0.06 bpm for STFT and −0.2 bpm for HFT were computed, which were not significant compared to the IQR. According to these results, the best performances were obtained for a window duration of 50 s. 

#### 3.2.3. Inter Subject Variability

**Figure 7:**
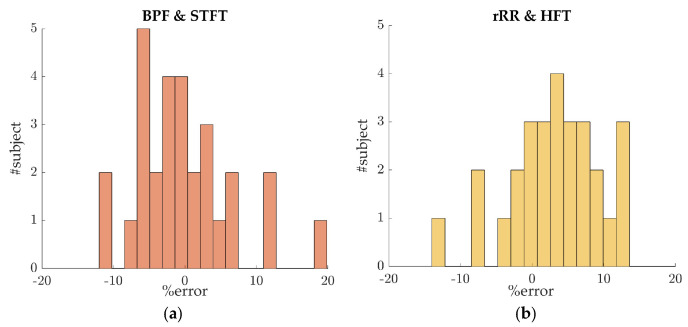
Percent error distributions for inter-subject variability evaluation. For each subject, the mean percent error was computed. In this plot, the data were averaged across a 50 s time-window: (**a**) error distribution for the algorithms BPF + STFT; (**b**) error distribution for the algorithms rRR + HFT.

The previous sections present the results obtained by pooling the error values of data from all athletes, with about 30 values per athlete depending on the window size, giving the overall bias and precision of the tested algorithms. The 95% CI values demonstrate the presence of large errors, thus suggesting the results to be highly subject dependent. In order to further explore this hypothesis, the mean percent error was computed for each subject, leading to 29 error values (Figure 7a,b). Figure 7 shows that 24 out of 29 and 16 out of 29 subjects had an overall mean percent error, during the incremental running test, that was lower than ±10% and ±5%, respectively. The remaining athletes (5 out of 29) presented high errors, which calls the persistence of RSA throughout the incremental test into question. 

## 4. Discussion

### 4.1. Validation of the BR Estimation Algorithms

The purpose of the present study was to test the accuracy of different BR estimations obtained from the PolarH10 as compared to the Cosmed metabolic measurement system. The originality of this work was the analysis of RSA during high intensity exercises such as running, as well as the testing and validation of algorithms that were only developed for resting assessments. 

**Figure A4:**
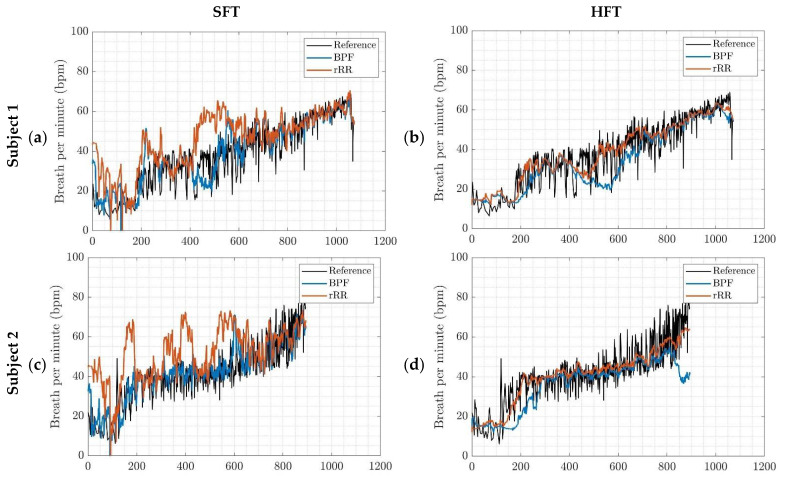

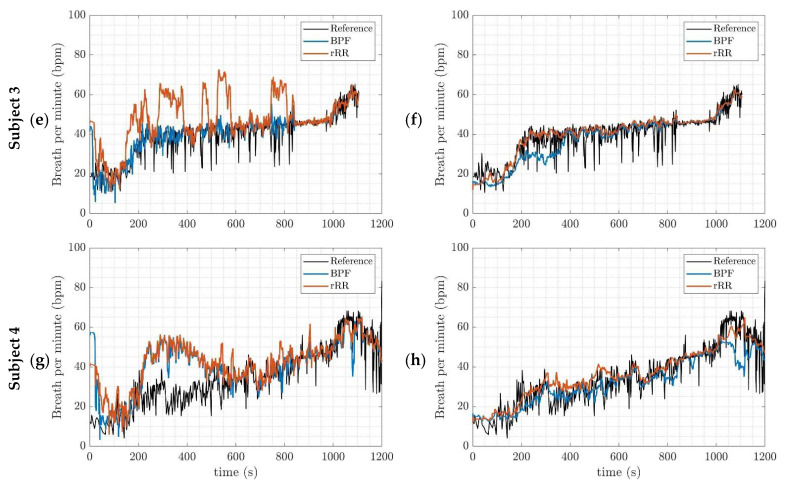
BR estimation, outcomes of the implemented algorithms for four different subjects; (**a**,**c**,**e**,**g**) SFT: singular frequency tracking; (**b**,**d**,**f**,**h**) HFT: harmonic frequency tracking. BPF: band-pass filter; rRR: relative RR intervals transformation.

Based on the current results, we suggest using BPF as preprocessing method for extracting respiratory signal from RR intervals. The rRR is also an appropriate pre-processing method when combined with the HFT algorithm. The results obtained with rRR reveal a high positive bias and IQR for all combinations, except when HFT was used. This suggests that rRR transformation might enhance frequency oscillations, including the fundamental and harmonics, thereby allowing HFT to efficiently track the frequency component of interest through a weighting procedure of harmonic components. In Appendix E, the STFT spectrograms of the filtered ${\mathrm{RR}}_{i}$ time-series show an increase in the frequency component, in contrast to the results obtained when rRR was used. This is observed both for the frequency of interest (corresponding to BR) and its harmonics, which explains the improved performances (IQR and correlation) when HFT was used. In Figure 4a,b, we can observe this phenomenon for one participant, as well as in Appendix D for additional subjects. Indeed, ${\mathrm{BR}}^{\mathrm{hr}}$ estimations using rRR + SFT and rRR + STFT sometimes have a tendency to track only the first harmonic (Figure A4, Appendix D), which is corrected when using HFT (Figure A4, Appendix D). An important observation, from our study conducted on non-stationary data collected while subjects ran at increasing speed, is that BPF + STFT provides the best results in term of bias and precision, and rRR + HFT provides the best results in terms of correlation. The Bland–Altman plot showed an overall bias of −0.08 bpm, and a 95% CI between 19.1 and −18.2 bpm for BPF + STFT (Figure 5a), and an overall bias of 1.1 bpm and a 95% CI between 16.4 and −12.2 bpm for rRR + HFT (Figure 5b).

Unsurprisingly, the performances of our algorithms when applied to running data were lower than the BR measured from ${\mathrm{RR}}_{i}$ at rest or directly from respiratory devices. Charlton. et al., 2016, in their systematic review on BR estimation from ECG and PPG, at rest reported an overall bias of 0.0 bpm and a 95% LOA of −4.7 to 4.7 bpm when time-domain breath-detection techniques were used [27]. Interestingly, our results suggest that frequency-based methods are more appropriate than time-based when processing RR intervals during running. Our hypothesis is that the amplitude of the respiratory signal extracted from RR intervals during exercises is lower and more variable than in resting condition, leading to higher errors in local maxima/minima peak detection. To the best of our knowledge, only Lee et al., 2011, extracted BR from ECG and PPG during high intensity treadmill running. The authors reported slightly better results when BR estimates were averaged across a 60 s time-window for a breathing frequency range of 0.8–1.0 Hz. The good performances of their algorithms might be explained by the processing of raw ECG using the fusion of amplitude and frequency modulations, which was shown to improve BR estimation [27,28]. Furthermore, previous studies reported higher correlation values when BR was directly measured from respiratory devices. A r-value of 0.93 was obtained for a portable respiratory inductive plethysmograph (RIP, Lifeshirt™, Vivometrics, Ventura, CA, USA), with pneumotachograph as the reference system, at different exercise intensities (walking to running 8.9 km/h) [14]. Another study tested the accuracy of a capacitive sensor (ZephyrTM BioHarness) versus a validated portable metabolic system (Model K4b, COSMED, Rome, Italy) and reported high correlation coefficients (r = 0.90–0.99) above 30% VO2max [15]. 

However, the BPF + STFT and rRR + HFT methods used in this study may help to provide reliable information on respiratory frequency from only heart rate monitoring with acceptable accuracy, less than 10%, during high intensity exercises such as running. 

### 4.2. Physiological Implications

According to the state-of-the-art research, accurate data about RSA under exercise conditions are scarce. Our results showed that, for a majority of our participants, the dynamic pattern of the frequency of RSA can be extracted from ${\mathrm{RR}}_{i}$ time-series obtained during an incremental running test. In agreement with previous studies, during exercise, RSA and breathing dynamically developed at the same frequency [20]. However, the results obtained for certain participants (5 out of 29) present high errors, which call the persistence of RSA throughout the incremental test into question. Therefore, a quality index evaluating the presence of RSA should be developed before generalizing the methodology to individual runners. Interestingly, the spectrograms provided in Appendix E support previous findings demonstrating that RSA decreases at low and medium workloads (~<62% VO2peak), and persists and increases at high and peak exercise workloads [20]. The exact physiological mechanisms involved in RSA modulation during sport is still under debate in the research field. It was shown previously that breathing affects the cardiovascular system, either directly (mechanical effects) or indirectly (neurally mediated effects). At rest, RSA is mainly influenced by vagal activity and can be used as a vagal tone index. It is well-known that vagal influence on heart rate variability is reduced during exercise [39,40,41]. Moreover, sympathetic contribution to our observed RSA is unlikely because the sympathetic nervous system responds too slowly to mediate heart rate fluctuations in very high respiratory frequency range [42]. The alternative hypothesis suggested by Blain et al., 2005, explaining the existence of RSA during running, is the enhancement of a non-neural mechanism [20]. Therefore, in response to the increase in ventilation, mechanical stretching of the sinus node is suggested to explain the persistence of RSA during high exercise intensity [22]. 

### 4.3. Perspective—Applications and Near-Real-Time Computation

Typically, BR might be used as a practical non-invasive method for estimating ventilatory thresholds during incremental exercise [5]. Then, it has also been shown that BR is closely associated with the subjects’ perceived exertion (RPE) during an activity [43], as well as being sensitive to different fatigue states [11,12]. Therefore, average BR may provide a simple preliminary description of the overall physical effort of a training session. Based on our knowledge, there is no consensus about the accuracy required for the BR metrics in sport applications. Nicolo et al., 2014, demonstrated a strong correlation between RPE and BR normalized to maximum BR value during 30 min continuous exercise and High Intensity Interval Training (HIIT) [43]. As the BR values are normalized by the maximum value, any systematic bias would not affect the result, as long as this bias does not depend on the BR range. In addition, the linear relationship between BR and RPE makes it possible to frame BR values according to the perspective of effort, with 80% of the BR maximum value corresponding to high perceived effort [43]. For the above-mentioned applications, the most important aspect is the dynamic changes of BR throughout the exercise and thecapability of accurately estimating the maximum BR value. Our results show that accuracy and precision do not depend on the BR range (Figure 5). In addition, the BPF + STFT and rRR + HFT algorithms are able to track very high intensities of up to 60–65 bpm (Appendix D). Furthermore, Kim et al., 2012, reported the BR values at different VO2 max states, showing changes of about 12–15% from 50 to 70%, 70 to 90% and 90% to VO2max. Thus, we can argue that 5% error is reasonable for detecting meaningful changes in this situation. A quality index would be of high interest to exclude segments with unreliable respiratory signal extracted from $\;{\mathrm{RR}}_{i}$, thereby ensuring that the error falls into the 5% range. 

For certain applications, a near-real time implementation providing feedback is relevant. The methods implemented are called instantaneous by definition. However, due to the nature of the computations, a tracking delay that is inherent to the algorithm exists. In the context of near-real-time computation, BPF or rRR pre-processing methods are suitable. For the BR estimation algorithms, STFT need to be implemented on a window of 2L + 1 for near-real-time computation, adding a delay of L (samples) to the output. Moreover, STFT is subject to compromised time-frequency resolution, because increasing the time resolution (e.g., reducing the output delay) negatively affects the frequency estimation. SFT, HFT and peak detection require a small sample size, thus minimizing the delay. Regarding the above-mentioned information, as well as our results, we suggest using the BFP + SFT or rRR + HFT methods in near-real time applications. In addition, we observed that the BR estimates from SFT and HFT are smoother than STFT or peak detection, making them appropriate candidates for near-real-time computation. Furthermore, the performances obtained for BPF + HFT are only slightly affected by the window durations, showing good results when averaged across 10 s time-windows, thus enabling a fairly small time delay. 

### 4.4. Other Algorithms

Based on the state-of-the-art and previous work, we decided to explore the performances of two pre-processing methods and four BR estimation algorithms, considering them as promising. However, we are aware that other algorithms could have been implemented. It is worth notifying in this section that we explored other algorithms that are not included in the main work. As explained in the introduction, Hilbert transform and Teager–Kaiser (TK) operator are commonly used for instantaneous frequency tracking. Their performances were tested on our database. However, we observed that although the BR waveforms obtained with the Hilbert transform and TK operator extract the frequency information of the RR intervals time-series under analysis, this information is cluttered with large spikes and negative values [44]. This phenomenon is observed in all subjects, and seems to mainly be caused by abrupt changes in the filtered ${\mathrm{RR}}_{i}$ signal. Therefore, we decided to exclude these two methods from further consideration in our work. In addition, based on Lee et al.’s work [45], we also identified the potential of singular spectrum analysis (SSA) decomposition as a pre-processing method. However, SSA decomposition is computationally costly and leads to slightly lower performances than BPF and rRR. Consequently, we also decided not to focus on this method. 

### 4.5. Limitations

The first limitation of this work might be linked to the measurement protocol. The HR monitoring and reference system were both manually started at the same time with two button clicks, which resulted in the systems not being exactly synchronized. However, we believe that if there is a small delay, it should not significantly affect the results, as we estimated the BR at time-windows from 10 to 60 s. In addition, we are aware that increasing the length of the time-window leads to a reduction in the number of data points per trial, consequently reducing the sample size for statistical computation. Sample sizes of about 3000 and 500 were obtained for time-windows of 10 s and 60 s, respectively. As the sample sizes were high (n > 100), we considered them to not have a significant effect on the computed error metrics. 

Another limitation is the subject dependent BR estimation accuracy, as observed in Section 3.2.3. A relative error lower than ±10% was obtained for a majority of the participants (23 out of 29), suggesting that the estimation is usable in practical applications. The reduction in RSA might explain the poor results obtained for the other subjects. A promising solution to overcome this issue is the development and validation of a quality index to estimate the quality of the respiratory signal extracted from ${\mathrm{RR}}_{i}$, as well as evaluating the persistence of RSA. 

Finally, another potential limitation might be the fact that when analyzing HRV, the blood pressure component might be present in addition to RSA component [46]. The measurement set-up does not allow us to reliably investigate this aspect that should be considered in future studies.

### 4.6. Future Work

We observed the subject-dependent performances of the BR estimations. The main reasons identified for this are as follows: (1) issues in the acquisition of RR intervals from ECG (measurements); and (2) loss of cardiorespiratory synchrony during exercises (physiology). As the accuracy of the PolarH10 sensor was validated during running [47], we would support the second hypothesis. Consequently, future work might focus on the physiology behind cardiorespiratory synchrony during high intensity exercises and the link to sports performance and fatigue. Indeed, the role of the vagal nerve at rest on sinus respiratory arrythmia (RSA) has been demonstrated. However, the exact mechanics explaining the synchrony during exercise remain unclear. 

Another interesting direction to further explore is the validation of a quality index to quantify the quality of the respiratory signal, named as filtered ${\mathrm{RR}}_{i}$ in this work, extracted from the ${\mathrm{RR}}_{i}$ frequency modulation [48]. A quality index can be used to detect artefacts in the RR intervals, as well as, more interestingly, to quantify the impact of a subject’s physiology on the heart rate data. Finally, the computational requirements of each algorithm were not investigated in this study. However, this may be important for use in near-real-time applications or for embedded software. In these settings, the precision and the computational cost are critical features that might strongly determine the choice of the algorithm. 

## 5. Conclusions

In conclusion, two methods (BPF + STFT and rRR + HFT) were found to provide reasonably accurate BR estimation over a wide range of breathing frequencies measured during a running VO2max test. Measurement of BR using in-field recorded data can be problematic, and our methodology offers a relatively unobtrusive manner of obtaining this information when an accuracy of more than 5.5% is not required. In addition, HR monitoring systems are widely used, thereby making our work attractive by allowing additional physiological insights without the need for an additional sensor. Finally, future challenges consist of applying/validating our approaches during in-field endurance running in the context of fatigue assessment.

## Figures and Tables

**Table 1:** Performances of the 8 combinations analyzed for the BR estimation from RR intervals.

		STFT	SFT	HFT	Peak
BPF	Bias (IQR) (bpm)	−0.08 (3.28)	1.31 (5.89)	−2.33 (8.73)	2.85 (8.27)
	CI (bpm), 2.5	−18.2	−13.91	−21.63	−8.66
	CI (bpm), 97.5	19.1	25.85	3.87	23.03
	MdAPE (%)	5.48	8.10	12.40	11.50
	ρ	0.82 *	0.77 *	0.81 *	0.80 *
rRR	Bias (IQR) (bpm)	2.35 (13.22)	7.30 (17.20)	1.06 (4.29)	9.18 (14.93)
	CI (bpm), 2.5	−7.92	−4.16	−12.24	−2.91
	CI (bpm), 97.5	30.51	31.22	16.46	32.14
	MdAPE (%)	11.24	23.81	7.66	30.16
	ρ	0.67 *	0.61 *	0.88 *	0.66 *

The BR estimation was averaged across the 50 s window duration. The table reports the bias, interquartile range (IQR), confidence interval (CI), median absolute percent error (MdAPE, median of *ϵ%*(w)) and Spearman’s correlation (ρ) of all 29 athletes pooled together (sample size: n = 609). BPF: band-pass filter; rRR: relative RR intervals transformation; STFT: short-term Fourier transform; STF: single frequency tracking; HFT: harmonic frequency tracking; Peak: peak detection method. * *p*-value lower than 0.05.

**Figure A1:**
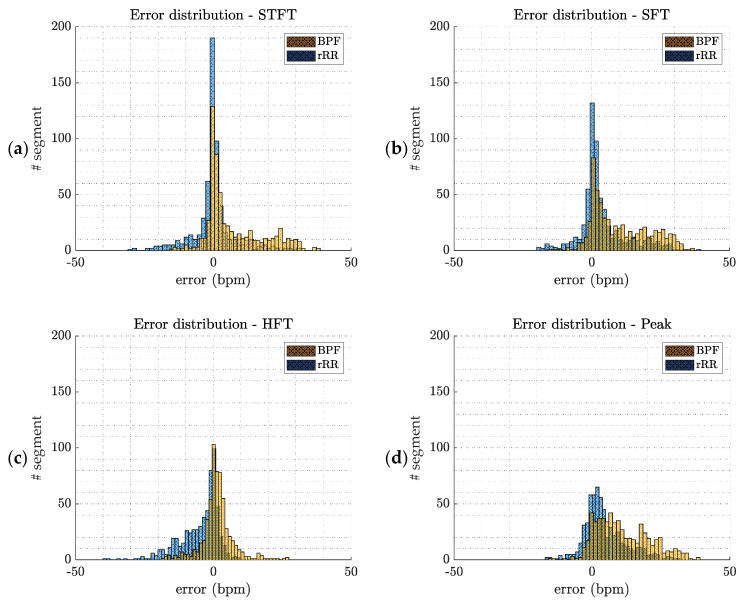
Error distributions (*ϵ*(w) $=\bar{{\mathrm{BR}}^{\mathrm{hr}}}\left(w\right)-\bar{{\mathrm{BR}}^{\mathrm{ref}}}\left(w\right),$ w: time-window) computed by pooling error values of all athletes. The two pre-processing methods are presented (BPF: band-pass filter; and rRR: relative RR intervals transformation); (**a**) short-term Fourier transform (STFT); (**b**) SFT: singular frequency tracking; (**c**) HFT: harmonic frequency tracking; (**d**) peak detection method.

**Figure A2:**
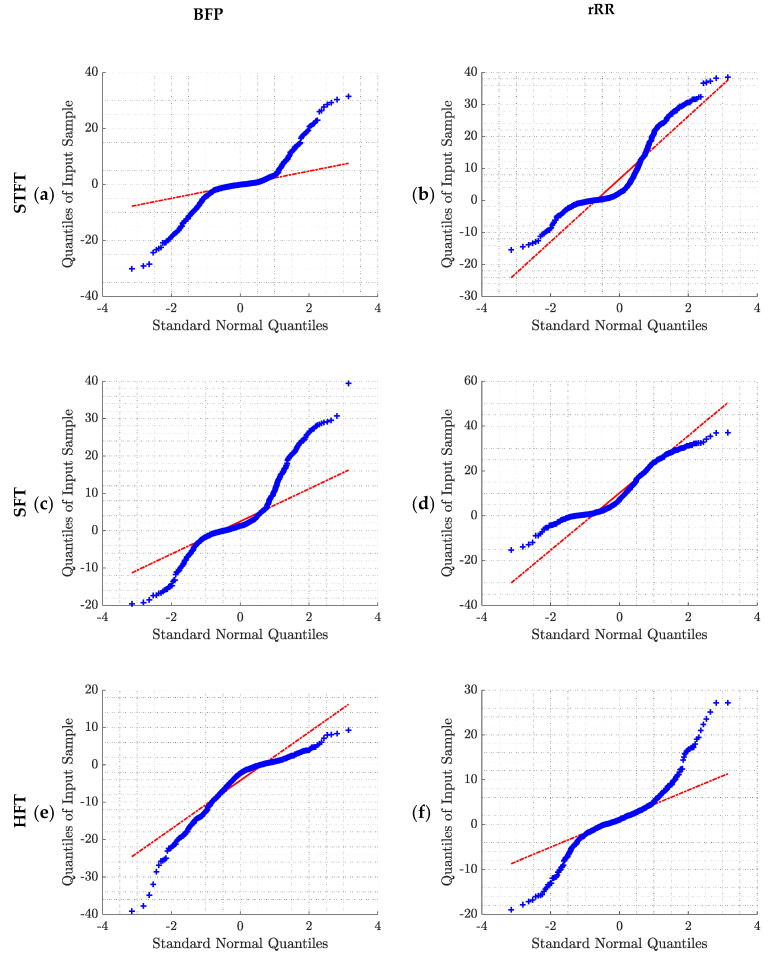

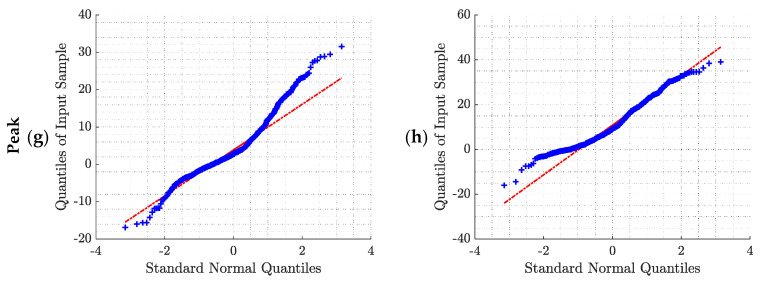
Q–Q plot of the error distributions obtained for the two pre-processing methods and the four BR estimation algorithms: (**a**) BPF + STFT; (**b**) rRR + STFT; (**c**) BPF + SFT; (**d**) rRR + SFT; (**e**) BPF + HFT; (**f**) rRR + HFT; (**g**) BPF + Peak; (**h**) rRR + Peak. BPF: band-pass filter; rRR: relative RR intervals transformation; STFT: short-term Fourier transform; SFT: single frequency tracker; HFT: harmonic frequency tracker; Peak: peak detection. The red line corresponds to the ideal scenario in case of normally distributed data.

**Figure A3:**
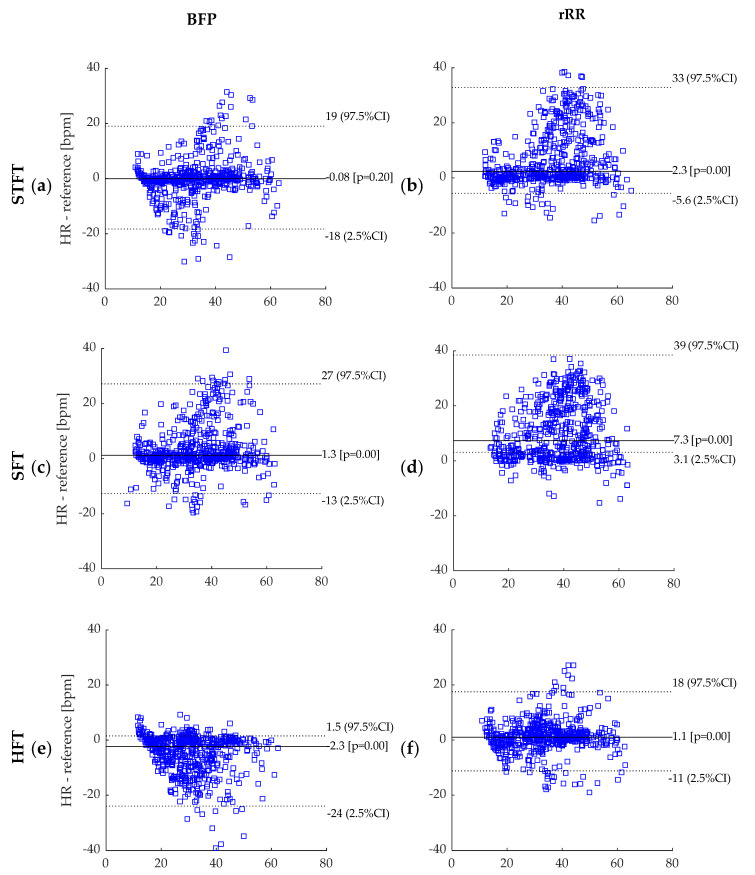

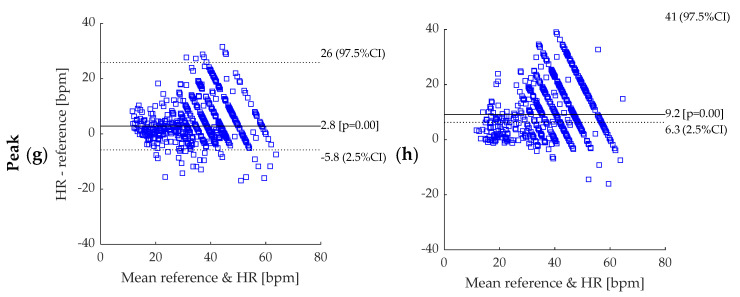
Bland–Altman plot: (**a**) BPF + STFT; (**b**) rRR + STFT; (**c**) BPF + SFT; (**d**) rRR + SFT; (**e**) BPF + HFT; (**f**) rRR + HFT; (**g**) BPF + Peak; (**h**) rRR + Peak. BPF: band-pass filter; rRR: relative RR intervals transformation; STFT: short-term Fourier transform; SFT: single frequency tracker; HFT: harmonic frequency tracker; Peak: peak detection. Dotted lines represent 95% CI and solid line represents the bias (bpm). The reference system indicated in the figures corresponds to the Cosmed Quark CPET measurement device used as ground truth.

**Figure A5:**
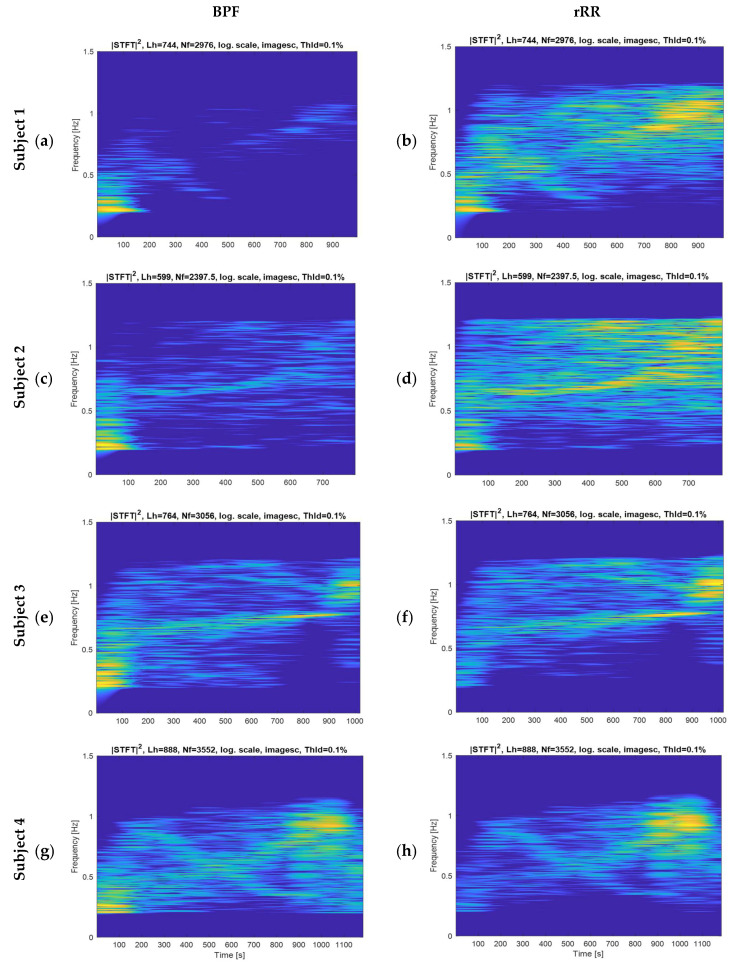
Spectrograms of the RR interval time-series pre-processed for four different subjects; (**a**,**c**,**e**,**g**) band-pass filter (BPF); (**b**,**d**,**f**,**h**) rRR: relative RR intervals transformation. Nf: number of frequency bins, corresponding to the length of the signal. Lh: frequency smoothing window.

## Data Availability

3rd Party Data; Restrictions apply to the availability of these data. Data was obtained from the sport science institute of the university of Lausanne and are available from the authors with the permission of the sport science institute of the university of Lausanne.

## Appendix A. Error Distributions and Normality Tests

This appendix provides additional information about the error distributions and the tests used for normality checking. The Shapiro–Wilk test was used to test the null hypothesis that the error comes from a normal gaussian distribution. However, if the sample size is sufficiently large, this test may reject the null hypothesis even if it is too small to have practical significance. Thus, in addition, we decided to compute the Q–Q (quantile–quantile) plot for further investigation. The Q–Q plot is a graphical method for comparing two probability distributions, in which quantiles are plotted against each other. Thus, the quantiles of the distribution of interest are plotted against a standard normal quantile from a normal distribution. The plot follows a 45°-line (y = x) if the two distributions are identical. In our study, all the *p*-values computed with the Shapiro–Wilk test were lower than 0.05, rejecting the null hypothesis of normality. Moreover, the Q–Q plots (Figure A2) confirm the non-gaussian distributions of the errors.

## Appendix B. Statistical Analysis

Table A1 shows the performances of all possible combinations of pre-processing and BR estimation algorithms. The Shapiro–Wilk test and Q–Q plot demonstrated that the errors *ϵ*(w) were not normally distributed. Consequently, non-parametric statistical tests were used for evaluation. Table A1 lists the bias (median of the error *ϵ*(w)), interquartile range (IQR), and Spearman’s correlation (ρ) obtained for each combination when BR is averaged across 10, 20, 30, 40, 50 and 60 s window durations.

**Table A1:** Performances of the 18 combinations analyzed for the BR estimation from RR intervals.

			STFT	SFT	HFT	Peak
10 s	BPF	Bias (IQR) (bpm)	−0.01 (4.72)	1.36(8.64)	−2.63 (9.21)	2.31 (10.30)
		CI (bpm), 2.5	−21.17	−15.28	−22.98	−14.08
		CI (bpm), 97.5	24.69	28.99	5.85	27.03
		*ϵ**%*(w) (%)	8.06	11.21	13.20	13.94
		ρ	0.74 *	0.71 *	0.79 *	0.72 *
	rRR	Bias (IQR) (bpm)	1.81 (14.14)	7.41(18.78)	1.26 (5.57)	8.80 (16.64)
		CI (bpm), 2.5	−10.75	−6.01	−14.21	−5.87
		CI (bpm), 97.5	32.48	32.98	16.47	33.04
		*ϵ**%*(w) (%)	13.09	24.47	9.97	27.41
		ρ	0.65 *	0.57 *	0.86 *	0.62 *
20 s	BPF	Bias (IQR) (bpm)	−0.07 (3.96)	1.10 (7.96)	−2.45 (9.01)	2.59 (9.14)
		CI (bpm), 2.5	−19.72	−14.24	−22.92	−11.37
		CI (bpm), 97.5	23.01	27.52	5.09	24.57
		*ϵ**%*(w) (%)	6.52	9.83	11.97	12.93
		ρ	0.77 *	0.71 *	0.80 *	0.76 *
	rRR	Bias (IQR) (bpm)	2.08 (13.54)	7.07 (18.51)	1.20 (4.94)	8.88 (15.67)
		CI (bpm), 2.5	−9.34	−4.71	−13.43	−4.52
		CI (bpm), 97.5	31.71	32.13	16.27	32.58
		*ϵ**%*(w) (%)	12.04	22.36	8.92	27.88
		ρ	0.65 *	0.58 *	0.87 *	0.64 *
30 s	BPF	Bias (IQR) (bpm)	−0.08 (3.80)	1.09 (7.68)	−2.51 (8.76)	2.29 (8.53)
		CI (bpm), 2.5	−19.42	−14.11	−22.74	−9.78
		CI (bpm), 97.5	21.90	28.14	4.89	23.34
		*ϵ**%*(w) (%)	6.56	9.35	12.02	11.69
		ρ	0.79 *	0.71 *	0.80 *	0.78 *
	rRR	Bias (IQR) (bpm)	2.00 (13.07)	7.57 (18.63)	1.13 (4.65)	9.01 (15.44)
		CI (bpm), 2.5	−9.33	−4.67	−13.06	−3.77
		CI (bpm), 97.5	31.10	31.73	16.36	32.47
		MdAPE (%)	11.41	23.22	8.38	27.06
		ρ	0.66 *	0.57 *	0.88 *	0.65 *
40 s	BPF	Bias (IQR) (bpm)	−0.08 (3.48)	1.06 (7.04)	−2.34 (8.81)	2.64 (8.43)
		CI (bpm), 2.5	−17.81	−13.51	−21.95	−9.35
		CI (bpm), 97.5	19.65	26.03	4.61	21.66
		MdAPE (%)	5.98	8.14	11.88	11.85
		ρ	0.82 *	0.74 *	0.80 *	0.79 *
	rRR	Bias (IQR) (bpm)	1.92 (12.82)	7.09 (18.23)	1.06 (4.47)	8.88 (15.27)
		CI (bpm), 2.5	−15.99	−21.28	−12.30	−3.59
		CI (bpm), 97.5	21.51	28.22	15.92	32.24
		MdAPE (%)	10.54	22.93	7.97	28.57
		ρ	0.68 *	0.60 *	0.88 *	0.65 *
	BPF	Bias (IQR) (bpm)	−0.08 (3.28)	1.31 (5.89)	−2.33 (8.73)	2.85 (8.27)
50 s		CI (bpm), 2.5	−18.2	−13.91	−21.63	−8.66
		CI (bpm), 97.5	19.1	25.85	3.87	23.03
		MdAPE (%)	5.48	8.10	12.40	11.50
		ρ	0.82 *	0.77 *	0.81 *	0.80 *
	rRR	Bias (IQR) (bpm)	2.35 (13.22)	7.30 (17.20)	1.06 (4.29)	9.18 (14.93)
		CI (bpm), 2.5	−7.92	−4.16	−12.24	−2.91
		CI (bpm), 97.5	30.51	31.22	16.46	32.14
		MdAPE (%)	11.24	23.81	7.66	30.16
		ρ	0.67 *	0.61 *	0.88 *	0.66 *
60 s	BPF	Bias (IQR) (bpm)	−0.08 (3.56)	1.05 (6.08)	−2.36 (8.88)	2.65 (8.01)
		CI (bpm), 2.5	−17.56	−13.66	−21.49	−6.66
		CI (bpm), 97.5	19.79	25.68	3.97	21.76
		MdAPE (%)	5.50	8.30	11.05	11.38
		ρ	0.81 *	0.77 *	0.81 *	0.81 *
	rRR	Bias (IQR) (bpm)	1.99 (13.04)	6.92 (17.28)	1.05 (4.58)	9.47 (15.56)
		CI (bpm), 2.5	−8.27	−3.77	−12.20	−2.61
		CI (bpm), 97.5	30.00	30.56	16.16	31.83
		MdAPE (%)	10.34	22.97	8.26	29.49
		ρ	0.67 *	0.61 *	0.88 *	0.66 *

The BR estimation computed on window-sizes of 10, 20, 30, 40, 50 and 60 s. The table reports the bias, interquartile range (IQR), confidence interval (CI), median absolute percent error (MdAPE, median of *ϵ%*(w)) and Spearman’s correlation (ρ) of all 29 athletes pooled together (sample size: n = 609). BPF: band-pass filter; SSA: singular spectrum analysis decomposition; rRR: relative RR intervals transformation; SFT: singular frequency tracking; HFT: harmonic frequency tracking. * *p*-value lower than 0.05.

## Appendix C. Bland–Altman Plots

This appendix provides the Bland–Altman plots obtained for the eight BR estimations algorithms when BR was averaged across a 50 s time-window. As the distributions were non-gaussian, bias was computed using median and 95% confidence interval (CI) using $\;2.5{\mathrm{th}}^{}$–$97.5{\mathrm{th}}^{}\;$ percentiles. The sample size (n = 609) corresponds to error values computed on 50 s time-window and pooled data from all athletes.
